# Red blood cell omega-3 fatty acids and attention scores in healthy adolescents

**DOI:** 10.1007/s00787-022-02064-w

**Published:** 2022-08-12

**Authors:** Ariadna Pinar-Martí, Silvia Fernández-Barrés, Florence Gignac, Cecilia Persavento, Anna Delgado, Dora Romaguera, Iolanda Lázaro, Emilio Ros, Mònica López-Vicente, Jordi Salas-Salvadó, Aleix Sala-Vila, Jordi Júlvez

**Affiliations:** 1 Unversitat Pompeu Fabra, Barcelona, Catalonia Spain; 2 Barcelona Institute for Global Health (ISGlobal), Barcelona, Catalonia Spain; 3 CIBER Epidemiología Y Salud Pública, Instituto de Salud Carlos III, Madrid, Spain; 4 Instituto de Investigación Sanitaria Illes Balears, Hospital Universitari Son Espases, Palma, Spain; 5 CIBER Fisiopatología de La Obesidad Y Nutrición, Instituto de Salud Carlos III (ISCIII), Madrid, Spain; 6 Cardiovascular Risk and Nutrition, IMIM (Hospital del Mar Medical Research Institute), Barcelona, Spain; 7 Lipid Clinic, Endocrinology and Nutrition Service, Hospital Clínic, Biomedical Research Institute August Pi I Sunyer (IDIBAPS), Barcelona, Spain; 8 Department of Child and Adolescent Psychiatry and Psychology, Erasmus MC University Medical Center, Rotterdam, the Netherlands; 9 Institut d’Investigació Sanitària Pere Virgili (IISPV), Hospital Universitari Sant Joan de Reus, Reus (Tarragona), Catalonia Spain; 10 Departament de Bioquímica i Biotecnologia, Unitat de Nutrició Humana, Universitat Rovira i Virgili, Reus, Spain; 11 The Fatty Acid Research Institute, Sioux Falls, SD USA

**Keywords:** Polyunsaturated fatty acids, Omega-3, Docosahexaenoic acid (DHA), Alpha-linolenic acid (ALA), Attention, Adolescence

## Abstract

Omega-3 fatty acids are critical for brain function. Adolescence is increasingly believed to entail brain vulnerability to dietary intake. In contrast to the abundant research on the omega-3 docosahexaenoic acid (DHA) in cognition, research on DHA and attention in healthy adolescents is scarce. In addition, the role of alpha-linolenic acid (ALA), the vegetable omega-3 fatty acid, is unexplored. We examined associations between DHA and ALA and attention function among a healthy young population. In this cross-sectional study conducted in 372 adolescents (13.8 ± 0.9 years-old), we determined the red blood cell proportions of DHA and ALA by gas chromatography (objective biomarkers of their long-term dietary intake) and measured attention scores through the Attention Network Test. We constructed multivariable linear regression models to analyze associations, controlling for known confounders. Compared to participants at the lowest DHA tertile (reference), those at the highest DHA tertile showed significantly lower hit reaction time-standard error (higher attentiveness) (28.13 ms, 95% confidence interval [CI] =  – 52.30;  – 3.97), lower hit reaction time ( – 38.30 ms, 95% CI =  – 73.28;  – 3.33) and lower executive conflict response ( – 5.77 ms, 95% CI =  – 11.44;  – 0.09). In contrast, higher values were observed in those at the top tertile of ALA in hit reaction time compared to the lowest one (46.14 ms, 95% CI = 9.90; 82.34). However, a beneficial association was observed for ALA, with decreasing impulsivity index across tertiles. Overall, our results suggest that DHA (reflecting its dietary intake) is associated with attention performance in typically developing adolescents. The role of dietary ALA in attention is less clear, although higher blood levels of ALA appear to result in lower impulsivity. Future intervention studies are needed to determine the causality of these associations and to better shape dietary recommendations for brain health during the adolescence period.

**Supplementary Information:**

The online version contains supplementary material available at 10.1007/s00787-022-02064-w.

## Introduction

Brain undergoes substantial structural and functional changes during adolescence (10 to 19 years of age, as defined by the World Health Organization) [1]. Some of the most relevant changes are observed in the prefrontal cortex (PFC) [1], which plays a key role in the ability to switch attentional control in response to changing task demands [2]. Attention is a complex cognitive function that precedes other high cognitive functions, such as decision-making, impulse control and strategic thinking [3]. The prominence of PFC in brain function is associated with a very large energetic demand, in particular during early life [4]. This is the basis for the belief that adolescence might entail brain vulnerability to dietary intake.

Polyunsaturated fatty acids (PUFAs) are critical for brain development and function [5], and their deficiency may have long-term functional consequences, such as memory impairment, attention-deficit hyperactivity disorder (ADHD), depression or anxiety disorders [5, 6]. Docosahexaenoic acid (C22:6n-3, DHA) is the most abundant PUFA in the brain [6]. DHA accrues rapidly in the PFC from the perinatal period to the first 18 years of life, with little increase after the second decade of life [7]. This suggests that the adolescent years are a crucial period to ensure adequate accrual of DHA in the PFC. In mammals, de novo synthesis of DHA is extremely inefficient; hence, DHA status in body tissues is directly related to dietary intake of DHA [8], which is mostly supplied by fatty fish.

Despite the fair grounds to surmise that DHA intake (or consumption of its parent food fatty fish) during adolescence might translate into long-lasting brain benefits, research specifically focusing on DHA and attention function in healthy adolescents is scarce. In fact, the majority of studies often include younger children (birth–10 years old) [9] or adolescents with ADHD [10]. Intake of DHA and eicosapentaenoic acid (C20:5n-3, EPA, another omega-3 fatty acid of marine origin) was found to relate to impulse control and function of the dorsal anterior cingulate gyrus in a cross-sectional study of 87 typically developing adolescents (11–13 years old) [11]. In a study conducted in 266 adolescents (13–15 years), those with higher blood EPA + DHA performed better in the attention function test [12]. Finally, marginal benefits were observed in attention performance after 12 weeks of fatty fish-based meals (3 times per week) compared to similar meals with omega-3 supplements [13]. In addition, the role of alpha-linolenic acid (C18:3n-3, ALA, the vegetable omega-3 essential fatty acid) has received little attention to date, perhaps because ALA has long been believed to merely act indirectly via marginal conversion to DHA [8]. However, the issue of whether ALA promotes brain health on its own remains to be elucidated. This is a relevant point, considering the low customary fish consumption in most Western societies. Further, ALA has been suggested to have neuroprotective effects, being involved in the promotion of neurogenesis and neuronal survival [14, 15].

In this cross-sectional study, we hypothesized that higher dietary intake of DHA and ALA would relate to better attention performance in healthy adolescents. To address this issue, we determined the proportion of ALA and DHA in red blood cells (RBCs), which is an objective and valid surrogate of their long-term dietary intake [16], and searched for associations with the Attention Network Test (ANT), which assesses the three attentional networks (orienting, alerting and executive attention) [17] and examines sustained attention based on reaction times (RT) to target stimuli [18].

## Materials and methods

### Study design and sample

This is a cross-sectional study using baseline data from the Walnuts Smart Snack Dietary Intervention Trial, a randomized controlled trial aimed to evaluate whether dietary supplementation with four walnuts per day (30 kernel grams) for 6 months improved brain neuropsychological and socio-emotional development compared to a control group (abstention from walnuts) in healthy teenagers (11–16 years old) [19]. We recruited 771 participants over a 12-month period (2015–2016) in 11 high schools of Barcelona evenly distributed in geographical terms. In the selection of centers, we aimed to include at least one high school per city district, while inviting public and private schools. Before randomization, at baseline, we asked participants to undergo several neuropsychological tests and provide information on their lifestyle. We asked a randomly selected subsample of participants (*n* = 372) to provide a blood sample to measure RBC omega-3 status. The eligible participants in the present study were those with fully available data on blood omega-3 status and neuropsychological tests (*n* = 332).

### Sociodemographic, clinical, and lifestyle data

An experienced fieldwork technician with the help of two more experts recruited participants in collaboration with the Barcelona school system. We first obtained permission from the boards of several schools in Barcelona to inform them about the trial. Then, we provided the schools with in-person description of the project (general school meetings with the families and at the classrooms with the pupils) and a recruitment flier to assess willingness to collaborate and grant us permission to contact interested families by telephone. Families interested in participating contacted us through the school and filled out a form with their telephone number. Trained personnel contacted the parents and explained the study in more detail.

At baseline, in a face-to-face visit, a fieldwork technician administered questionnaires to the adolescent participants, while other questionnaires were given to be filled up at home by the parents and returned to us through the school teachers. We obtained data from parental education and occupation (from which the occupational social class was constructed, categorized as manual or non-manual [20]), parental mental health status, sociodemographic characteristics, adolescent health history, and lifestyle habits such as physical activity, sleep duration, and alcohol or drug use (collected as consumption of substances at any point in time). The technician also inquired about consumption of supplements and vitamins, and dietary habits through a food-frequency questionnaire (FFQ) of 60 food items, validated for the Spanish population [21] and adapted to the adolescent range. We calculated the weekly consumption of food groups, including consumption of fatty fish (constructed from the sum of all FFQ items related to seafood consumption: fatty fish, white fish, canned fish and fish by-products) and nuts (walnuts, almonds, hazelnuts and others). We also assessed the adherence to the Mediterranean diet by creating a score based on the consumption of fruits, vegetables, legumes, seafood, cereals, nuts, dairy, and olive oil (kidmed test) [19, 22, 23]. Data were obtained from a short true/false 16-item questionnaire from the FFQ modified to a relative score, with a final total score of 12. A score of 3 or less equals low adherence, a score from 4 to 7 equals medium adherence and a score of 8 or greater equals high adherence. Due to a very small portion of participants with low adherence (*n* = 18), the variable was reclassified in two categories (1–7 = low adherence; 8–12 = high adherence). Height, weight, and waist circumference was measured by standard methods, and based on weight /height^2^ body mass index (BMI) was calculated.

A nurse obtained blood samples after an overnight fast. We centrifuged the samples at 2500 g for 20 min at 20ºC within 4 h after extraction. We obtained a 1500-μL aliquot of packed RBCs, which was stored at  – 80 °C until fatty acid analysis. We determined the fatty acid profile in RBC by gas chromatography, as described [24]. In brief, cells contained in a 100-μL aliquot of RBCs were hemolyzed and spun. The pellet membranes were dissolved in 1 mL BF_3_ methanol solution and heated to hydrolyze and methylate glycerolphospholipid fatty acids contained in the RBC membrane. The fatty acid methyl esters were isolated by adding n-hexane and were separated by gas chromatography using an Agilent HP 7890 Gas Chromatograph equipped with a 30 m × 0.25 μm × 0.25 mm SupraWAX-280 capillary column (Teknokroma, Barcelona, Spain), an autosampler, and a flame ionization detector. The amount of each fatty acid was expressed as a percentage of the total identified fatty acids in the sample. The PUFA included in this study are DHA and ALA. Participants were classified based on tertiles of each fatty acid (from the lowest to the highest concentration, divided proportionally to sample size).

### Attention function assessment

Several computer-based neuropsychological tests were assessed at the schools following a strict protocol and with the supervision of an experienced psychologist. Schoolrooms satisfying optimal conditions for testing were previously selected and the fieldwork technicians prepared the laptops and related equipment. One-hour sessions were conducted in groups of 15 adolescents by three examiners supervised by the psychologist, who explained the test instructions before starting each session.

To determine the attention function performance, we used the Attention Network Test (ANT). The ANT is a valid computer-based test aimed to assess the three attentional networks (orienting, alerting and executive attention) [17] and examine sustained attention based on reaction times (RT) to target stimuli [18]. The test consisted of the appearance of five arrows on the screen and participants had to indicate the direction of the central arrow as fast as possible. A detailed description of ANT can be found elsewhere [18]. The main ANT outcomes selected were hit reaction time (HRT) and hit reaction time standard error (HRT-SE), which measure median RT and standard error of RT for correct responses, respectively, both being indicators of selective and sustained attention; conflict response (executive attention), measured as the subtraction of RT in the absence of conflict from the RT in the presence of conflict; and impulsivity, measured as the subtraction of RT in incorrect responses from the RT in correct responses. All outcomes are measured in milliseconds. Lower scores indicate better attention performance in all measures. Additional measures on attention outcomes, such as the alerting and the orienting network, were included in the supplementary material.

### Statistical analyses

Associations between selected RBC PUFA and attention scores were evaluated using multivariable linear regression models. PUFA levels were evaluated as ordinal variables (tertiles). ANT outcomes were evaluated as continuous variables. For all analyses, two models were built between the exposure and the outcomes: first, a minimally adjusted model with some mandatory covariates, i.e., sex, age, and center (school); secondly, a fully multivariable model with the main confounders included. The main confounders were selected according to previous scientific knowledge and then building up a Directed Acyclic Graph (DAG) model (Fig. S1). Fully adjusted models included BMI, alcohol consumption (yes/no), smoking habits (yes/no), physical activity (≥ 3 times/week, yes/no), maternal and paternal educational level (university studies/lower education), maternal and paternal occupational social class (manual/non-manual), and the modified relative Mediterranean diet score (low/high adherence). Analyses were limited to adolescents with complete information on the variables included in the models.

Additionally, we carried out a supplementary analysis to compare DHA and ALA RBC levels between participants reporting (i) consumption of at least four weekly servings of fatty fish versus lower consumption; and (ii) consumption of at least one weekly serving of nuts versus lower consumption, two cut-offs selected based on statistical criteria to evaluate if self-reported consumption-matched blood PUFA levels. To further explore the association between selected RBC omega-3 PUFA and attention performance, we conducted supplementary multivariable linear regression analyses with the rest of the attentional network outcomes (orienting and alerting). Although gender differences are important in the stages of cognitive development during adolescence, it was not possible to carry out the statistical models stratified by gender due to the small size of the sample although gender was used as a mandatory covariate to adjust all models.

All statistical analyses were conducted using STATA 13. Two-tailed *p* values below 0.05 were considered statistically significant.

## Results

The main characteristics of the study population are shown in Table 1 (extended information can be found in Table S1). Participants had an equal gender distribution, age was within a few years of rank (mean, 13.8 years, SD = 0.9), and RBC DHA and ALA levels were within the expected range (mean of 3.96 and 0.11, respectively). RBC DHA status was significantly higher (*P* < 0.001) in participants reporting high consumption of fish (≥ 4 servings /week) compared to those reporting lower consumption. In contrast, no significant differences (*P* = 0.454) were observed for RBC ALA between those reporting high- and low nut consumption (Table S2).

**Table 1:** Characteristics of the study population sample (*n* = 332)

Variable	Value
*Adolescents*	
Female gender, *N* (%)	164 (49.40)
Age, years	13.84 ± 0.93
BMI, kg/m^2^	20.32 ± 3.23
Physical activity ≥ 3 times/week, *N* (%)	184 (59.35)
Alcohol consumer, *N* (%)^a^	133 (43.46)
Smoker, *N* (%)^b^	61 (19.81)
Higher adherence to Mediterranean diet, *N* (%)^c^	124 (40.66)
RBC C22:6n-3 (DHA, % of total fatty acids)	3.96 ± 0.84
RBC C18:3n-3 (ALA, % of total fatty acids)	0.11 ± 0.08
ANT HRT-SE, ms^d^	144.44 ± 75.40
ANT HRT, ms^d^	568.66 ± 111.28
Impulsivity Index, ms^d,e^	59.44 ± 155.67
Conflict response, ms^d^	67.32 ± 32.77
*Mother*	
University education, *N* (%)	210 (63.44)
Non-manual occupation, *N* (%)	248 (80.26)
*Father*	
University education, *N* (%)	188 (57.14)
Non-manual occupation, *N* (%)	211 (70.10)

Data are expressed as mean ± standard deviation, unless otherwise stated

*BMI* body mass index, *RBC* red blood cell, *ANT* attention network test, *HRT* hit reaction time, *SE* standard error, *m*s, milliseconds

^a^Defined as if they have ever consumed alcoholic beverages (yes/no)

^b^Defined as if they have ever smoked a cigarette, whether tobacco-based or electronic (yes/no)

^c^A high score equals a score ≥ 8 (of a total of 12)

^d^Higher scores indicate worse attention performance

^e^Data in *n *= 256 participants

Minimally and fully adjusted regression models for associations between DHA and ALA levels with the main attention outcomes are shown in Tables 2,3,4, and 5. Increasing RBC DHA was associated with a reduction of ANT HRT-SE in both minimally and fully adjusted models (Table 2). A significant linear trend across tertiles of RBC DHA was observed (P for trend = 0.024 for fully adjusted model). Compared to the lowest tertile, those at the highest tertile showed significantly lower hit reaction time standard error (adjusted mean,  – 28.13 ms, 95% CI =  – 52.30;  – 3.97; *P* = 0.046 and *P* = 0.024 for minimally and fully adjusted models, respectively). No significant associations were observed for RBC ALA. We also observed an inverse association between RBC DHA and ANT HRT (Table 3), although statistical significance was limited to the fully adjusted model (*P* for trend = 0.031), with lower reaction time for participants at the highest tertile compared to those at the bottom tertile (adjusted mean,  – 38.30 ms, 95% CI =  – 73.28;  – 3.33; *P* = 0.032). Of note, increasing values were observed across tertiles of RBC ALA in the fully adjusted model (P for trend = 0.013), with significantly higher values in those at the top tertile compared to the bottom tertile (adjusted mean, 46.14 ms, 95% CI = 9.90; 82.34; *P* = 0.013). Regarding impulsivity (Table 4), while no significant associations were observed for DHA, a negative association was observed for RBC ALA, with decreasing impulsivity indices across tertiles (*P* for trend = 0.079 and *P* = 0.042 for minimally and fully adjusted models, respectively). We also observed significant inverse associations for RBC DHA (but not RBC ALA) in conflict response or executive attention (Table 5), with participants in the lowest tertile showing significantly higher scores than those in either medium or highest tertile (*P* for trend = 0.009 and *P* = 0.046 for minimally- and fully-adjusted models, respectively). Finally, we observed no significant associations between the two fatty acids of interest and scores on orienting (Table S3) or alerting (Table S4) networks.

**Table 2:** Association between red blood cell proportions of C22:6n-3 (DHA) and C18:3n-3 (ALA) in tertiles and ANT scores on HRT-SE

Fatty acid^a^	ANT HRT-SE, ms							
	Minimally adjusted^b^				Fully adjusted^c^			
	*N*	Coef	(95% CI)	*P* value	*N*	Coef	(95% CI)	*P* value
C22:6n-3								
1st tertile (3.12)	106	Ref			76	Ref		
2nd tertile (3.89)	104	– 18.53	( – 38.54; 1.48)	0.069	84	– 20.90	( – 43.98; 2.17)	0.076
3rd tertile (4.75)	106	– 21.12	( – 41.83; -0.41)	0.046	88	– 28.13	( – 52.30; -3.97)	0.023
Tertiles in continuous^d^	316	– 10.63	( – 20.98; -0.28)	0.044	248	– 13.94	( – 26.01; -1.88)	0.024
C18:3n-3								
1st tertile (0.06)	104	Ref			85	Ref		
2nd tertile (0.09)	103	5.89	( – 15.55; 27.33)	0.589	89	20.68	( – 3.26; 44.61)	0.090
3rd tertile (0.14)	109	6.84	( – 15.55; 29.23)	0.548	74	20.84	( – 4.10; 45.78)	0.101
Tertiles in continuous^d^	316	3.33	( – 7.83; 14.49)	0.557	248	10.08	( – 2.38; 22.53)	0.112

*ANT* attention network test, *HRT* hit reaction time, *SE* standard error, *ms* milliseconds, *CI* confidence interval, *Ref* reference group, *Coef *β coefficients estimated by linear regression models, *N* number of subjects with available data

Higher scores indicate worse attention performance

^a^Median of each fatty acid within tertile category

^b^Adjusted for: school center, age and gender of the child

^c^Additionally adjusted for: body mass index of the child, alcohol consumption (have you ever consumed alcoholic beverages? yes/no), smoking habit (have you ever smoked a normal or electronic cigarette? yes/no), physical activity ≥ 3 times/week (yes/no), modified Mediterranean diet score (1–7 “low”/8–12 “high”), maternal educational level (university studies/lower education), paternal education level (university studies/lower education), maternal occupational social class (manual/non-manual) and paternal occupational social class (manual/non-manual)

^d^The *p* value is *P* for trend

**Table 3:** Association between red blood cell proportions of C22:6n-3 (DHA) and C18:3n-3 (ALA) in tertiles and ANT scores on HRT

Fatty acid^a^	ANT HRT, ms							
	Minimally adjusted^b^				Fully adjusted^c^			
	*N*	Coef	(95% CI)	*P* value	*N*	Coef	(95% CI)	*P* value
C22:6n-3								
1st tertile (3.12)	106	Ref			76	Ref		
2nd tertile (3.89)	104	– 14.56	( – 43.57; 14.46)	0.324	84	– 13.84	( – 47.59; 19.91)	0.420
3rd tertile (4.75)	107	– 27.73	( – 57.60; 2.14)	0.069	89	– 38.30	( – 73.28; -3.33)	0.032
Tertiles in continuous^d^	317	– 13.87	( – 28.78; 1.04)	0.068	249	– 19.27	( – 36.71; -1.82)	0.031
C18:3n-3								
1st tertile (0.06)	104	Ref			85	Ref		
2nd tertile (0.09)	104	9.57	( – 21.31; 40.45)	0.543	90	27.30	( – 7.50; 62.10)	0.124
3rd tertile (0.14)	109	26.24	( – 6.01; 58.49)	0.110	74	46.14	(9.90; 82.34)	0.013
Tertiles in continuous^d^	317	13.25	( – 2.82; 29.32)	0.106	249	22.93	(4.87; 40.98)	0.013

*ANT* attention network test, *HRT* hit reaction time, *ms* milliseconds, *CI* confidence interval, *Ref* reference group, *Coef *β coefficients estimated by linear regression models, *N* number of subjects with available data

Higher scores indicate worse attention performance

^a^Median of each fatty acid within tertile category

^b^Adjusted for: school center, age, and gender of the child

^c^Additionally adjusted for: body mass index of the child, alcohol consumption (have you ever consumed alcoholic beverages? yes/no), smoking habit (have you ever smoked a normal or electronic cigarette? yes/no), physical activity ≥ 3 times/week (yes/no), modified Mediterranean diet score (1–7 “low”/8–12 “high”), maternal educational level (university studies/lower education), paternal education level (university studies/lower education), maternal occupational social class (manual/non-manual) and paternal occupational social class (manual/non-manual)

^d^The *p* value is *P* for trend

**Table 4:** Association between red blood cell proportions of C22:6n-3 (DHA) and C18:3n-3 (ALA) in tertiles and ANT scores on impulsivity index

Fatty acid^a^	Impulsivity index, ms							
	Minimally adjusted^b^				Fully adjusted^c^			
	*N*	Coef	(95% CI)	*P* value	*N*	Coef	(95% CI)	*P* value
C22:6n-3								
1st tertile (3.12)	88	Ref			61	Ref		
2nd tertile (3.89)	82	24.12	( – 25.45; 73.69)	0.339	65	17.82	( – 45.35; 80.98)	0.578
3rd tertile (4.75)	82	18.02	( – 31.86; 67.90)	0.477	72	27.11	( – 35.86; 90.07)	0.397
Tertiles in continuous^d^	252	9.15	( – 15.76; 34.06)	0.470	198	13.42	( – 17.93; 44.78)	0.399
C18:3n-3								
1st tertile (0.06)	84	Ref			70	Ref		
2nd tertile (0.09)	85	4.67	( – 46.65; 55.99)	0.858	73	14.12	( – 48.27; 76.51)	0.656
3rd tertile (0.14)	83	– 48.90	( – 104.43; 6.63)	0.084	59	– 66.22	( – 131.80; – 0.65)	0.048
Tertiles in continuous^d^	252	– 24.86	( – 52.67; 2.94)	0.079	198	– 34.19	( – 67.16; – 1.22)	0.042

*ms* milliseconds, *CI* confidence interval, *Ref* reference group, *Coef.* β coefficients estimated by linear regression models, *N* number of subjects with available data

Higher scores indicate worse attention performance (higher impulsivity)

^a^Median of each fatty acid within tertile category

^b^Adjusted for: school center, age, and gender of the child

^c^Additionally adjusted for: body mass index of the child, alcohol consumption (have you ever consumed alcoholic beverages? yes/no), smoking habit (have you ever smoked a normal or electronic cigarette? yes/no), physical activity ≥ 3 times/week (yes/no), modified Mediterranean diet score (1–7 “low”/8–12 “high”), maternal educational level (university studies/lower education), paternal education level (university studies/lower education), maternal occupational social class (manual/non-manual) and paternal occupational social class (manual/non-manual)

^d^The *p* value is *P* for trend

**Table 5:** Association between red blood cell proportions of C22:6n-3 (DHA) and C18:3n-3 (ALA) in tertiles and ANT scores on conflict network

Fatty acid^a^	Conflict response or executive attention, ms							
	Minimally adjusted^b^				Fully adjusted^c^			
	*N*	Coef	(95% CI)	*P* value	*N*	Coef	(95% CI)	*P* value
C22:6n-3								
1st tertile (3.12)	106	Ref			76	Ref		
2nd tertile (3.89)	103	– 13.08	( – 22.12; – 4.03)	0.005	83	– 15.93	( – 26.82; – 5.03)	0.004
3rd tertile (4.75)	107	– 12.43	( – 21.72; – 3.14)	0.009	89	– 11.98	( – 23.24; – 0.71)	0.037
Tertiles in continuous^d^	316	– 6.25	( – 10.92; -1.59)	0.009	248	– 5.77	( – 11.44; – 0.09)	0.046
C18:3n-3								
1st tertile (0.06)	103	Ref			84	Ref		
2nd tertile (0.09)	104	– 0.85	( – 10.63; 8.95)	0.865	90	0.28	( – 11.22; 11.78)	0.962
3rd tertile (0.14)	109	– 2.84	( – 13.06; 7.38)	0.585	74	– 1.81	( – 13.79; 10.17)	0.766
Tertiles in continuous^d^	316	– 1.44	( – 6.53; 3.65)	0.578	248	– 0.95	( – 6.92; 5.02)	0.755

*ms*, milliseconds; *CI*, confidence interval; *Ref*, reference group; *Coef.*, β coefficients estimated by linear regression models; *N*, number of subjects with available data

Higher scores indicate worse attention performance

^a^Median of each fatty acid within tertile category

^b^Adjusted for: school center, age, and gender of the child

^c^Additionally adjusted for: body mass index of the child, alcohol consumption (have you ever consumed alcoholic beverages? yes/no), smoking habit (have you ever smoked a normal or electronic cigarette? yes/no), physical activity ≥ 3 times/week (yes/no), modified Mediterranean diet score (1–7 “low”/8–12 “high”), maternal educational level (university studies/lower education), paternal education level (university studies/lower education), maternal occupational social class (manual/non-manual) and paternal occupational social class (manual/non-manual)

^d^The *p* value is *P* for trend

## Discussion

In this cross-sectional study conducted within the framework of the Walnuts Smart Snack Dietary Intervention Trial [19], we observed that two RBC omega-3 fatty acids were associated with several attention outcomes in healthy Spanish adolescents. In fully adjusted multivariable models, higher RBC DHA proportions (reflecting consumption of fatty fish) were associated with better attention functioning, based on scores of shorter latencies of hit reaction time and lower executive conflict response. In contrast, our findings suggest a less clear role of dietary ALA in attention. Higher proportion of ALA in RBC was associated with increased latencies of hit reaction time, but with a lower impulsivity response. However, RBC ALA could not reflect dietary nut intake since RBC ALA levels did not correlate with higher consumption of nuts, a finding somehow expected considering that nuts other than walnuts are poor sources of ALA. Thus, the effect of RBC ALA in hit reaction time and impulsivity response cannot be explained by nut dietary intake.

Two findings merit to be highlighted. First, we suggest a plausible role of dietary DHA in selective and sustained attention and in detecting and resolving stimuli conflict (executive functioning) in attention tasks, a notion to be confirmed in future randomized controlled trials in healthy teenagers. Our results concur with those from another observational study using a similar approach [12], adding evidence on the brain benefits of fatty fish consumption (the main source of DHA) in this population segment, to date mostly related to cognitive performance [25–27]. Overall, this reinforces the long-known importance of ensuring an adequate DHA accumulation in periods of life when the brain is developing or evolving, being adolescence of paramount importance.

Second, to our knowledge, this is the first study exploring the association between ALA (the vegetable omega-3 fatty acid) and attention performance in healthy adolescents. Since many individuals avoid fish for different reasons (veganism, ethical issues, sensitivity towards environmental sustainability, and/or concern about mercury and other pollutants) and the growing pressure on fisheries to meet consumption demands, there is a need for dietary substitutes for marine-derived omega-3 PUFA, and ALA is a good candidate. Research on ALA in pediatrics is restricted to two randomized controlled trials in participants with attention deficit-hyperactivity disorder, reporting null benefits for ALA, either alone [14] or in combination with linoleic acid [15]. Interestingly, we observed opposite associations for ALA in sustained attention, with participants in the highest tertile of RBC ALA showing longer reaction time latencies compared to those at the lowest one. A plausible explanation for such a finding can be found in the impulsivity index, given that participants at higher levels of RBC ALA were those with lower impulsivity indexes (the lower the impulsivity, the longer the reaction time) [28]. This result might be of great clinical relevance, since impulsivity is known to be a key feature of several psychiatric disorders (i.e. ADHD, personality and substance abuse disorders, etc.) [29–31]. However, although there appears to be a positive relationship between higher RBC ALA levels and impulsivity, this association cannot be explained by diet-related factors (i.e., higher dietary nut intake). Thus, further research is warranted to explore whether ALA supplementation could have a beneficial effect in treatment plans for these disorders. Randomized controlled trials involving ALA supplementation in healthy populations, such as the Walnuts Smart Snack Dietary Intervention Trial [19] will hopefully shed light on this issue.

An important strength of our study is the use of computerized cognitive tests focused on attention-specific outcomes rather than using more abstract and complex measures, such as school performance scores, or teacher (or self-reported) rating scales based on more indirect measures of neurobehaviour. Correspondingly, the assessment of the attention network is an important and close enough proxy to learning and academic achievement, since it precedes higher cognitive functions such as memory or strategic thinking [17, 32]. A second strength is the fact that our exposures (dietary DHA and ALA) do not rely on a method involving self-reporting (i.e., 24-h recalls or food-frequency questionnaires), which have several limitations that affect both the accuracy and precision of the measurement (i.e., over- and under-reporting, interviewer bias, incorrect portion size estimations, coding and computational errors, and errors associated with the use of food composition databases). To avoid these caveats, we conducted lipidomics, determining the omega-3 status in RBCs. Because ALA is an essential fatty acid and de novo synthesis of DHA from ALA is marginal, both circulating ALA and DHA are increasingly used as objective biomarkers of their dietary intake [16]. Given the RBC lifespan (around 120 days), the omega-3 profile of RBC better reflects long-term intake than omega-3 in whole plasma or serum [16]. When exploring the association between the two exposures of interest (RBC status of ALA and DHA) and self-reported consumption of their parent foods, while RBC DHA levels were directly associated with self-reported fish consumption, no association was found between self-reported nut consumption and RBC ALA levels. This may be due to the low consumption of walnuts (the main nut source of ALA) among the participants, resulting in low RBC ALA levels in both those reporting more than one serving a week and those reporting less. Of note, the data presented in this study is part of a larger intervention study in which exclusion criteria included eating walnuts on a daily basis, which may be an explanation for the low consumption of nuts in the sample.

The main limitation of this study is that, given its cross-sectional nature, causality cannot be inferred and it is not possible to establish the directionality of associations. Additionally, because of observational design of this study, we could not exclude the possibility that uncaptured environmental factors may have influenced or caused the observed association. Finally, given the use of questionnaires to collect information on the possible confounders, sample size was significantly reduced in the fully adjusted models due to missing responses. However, we found significant associations despite a relatively small sample size.

Overall, our results suggest that dietary DHA is associated with attention performance in typically developing adolescents, specifically with selective and sustained attention, and detecting and resolving conflict. In contrast, dietary ALA cannot be associated with better attention performance, although higher levels of RBC ALA appear to have a positive effect on impulsivity. Intervention studies are needed to determine the causality of these associations and to further elucidate the effects of omega-3 PUFAs beyond cognitive performance during adolescence. This research is warranted to help better shape basic dietary recommendations for the adolescent population to ensure an optimal dietary omega-3 PUFA intake for a healthy brain development.

### Supplementary Information

Below is the link to the electronic supplementary material.

Supplementary file1 (PDF 271 KB)
