# Supplementary material for: Red blood cell omega-3 fatty acids and attention scores in healthy adolescents
Source: Eur Child Adolesc Psychiatry. 2022 Aug 12;32(11):2187–95. doi: 10.1007/s00787-022-02064-w (PMC10576734; doi:10.1007/s00787-022-02064-w)
Supplement: Supplementary file 1 — Supplementary file1 (PDF 271 KB) [file 787_2022_2064_MOESM1_ESM.pdf]

Supplementary Information

Red blood cell omega-3 fatty acids and attention scores in healthy adolescents

Ariadna Pinar-Martí[1,2], Silvia Fernández-Barrés PhD[1,2,3,], Florence Gignac MSc1,[2], Cecilia Persavento PhD[2], Anna Delgado PhD[2], Dora Romaguera PhD[2,4,5], Iolanda Lázaro PhD[6], Emilio Ros MD PhD[5,7], Mònica López-Vicente PhD[8], Jordi Salas-Salvadó MD PhD[5,9,10], Aleix Sala-Vila PhD[6,11*], Jordi Júlvez PhD[1,2,3,9,10*]

[1] Unversitat Pompeu Fabra, Barcelona, Catalonia, Spain

[2] Barcelona Institute for Global Health (ISGlobal), Barcelona, Catalonia, Spain

[3] CIBER Epidemiología y Salud Pública, Instituto de Salud Carlos III, Madrid, Spain

[4] Instituto de Investigación Sanitaria Illes Balears, Hospital Universitari Son Espases, Palma, Spain

[5] CIBER Fisiopatología de la Obesidad y Nutrición, Instituto de Salud Carlos III (ISCIII), Madrid, Spain

[6] Cardiovascular risk and nutrition, IMIM (Hospital del Mar Medical Research Institute), Barcelona, Spain

[7] Lipid Clinic, Endocrinology and Nutrition Service, Hospital Clínic, Biomedical Research Institute August Pi i Sunyer (IDIBAPS), Barcelona, Spain.

[8] Department of Child and Adolescent Psychiatry and Psychology, Erasmus MC University Medical Center, Rotterdam, the Netherlands.

[9] Institut d'Investigació Sanitària Pere Virgili (IISPV), Hospital Universitari Sant Joan de Reus, Reus (Tarragona), Catalonia, Spain

[10] Universitat Rovira i Virgili, Facultat de Medicina i Ciències de la Salut, Human Nutrition Unit, Reus, Spain.

[11] The Fatty Acid Research Institute, Sioux Falls, SD, USA

Target journal: European Child & Adolescent Psychiatry

Corresponding author:

Jordi Julvez (jordi.julvez@iispv.cat)

*JJ and ASV share equal contribution as senior authors.

Supplementary Figure 1. Directed Acyclic Graph (DAG) for investigating casual paths (possible confounders) between RBC PUFA levels and cognitive performance[a].

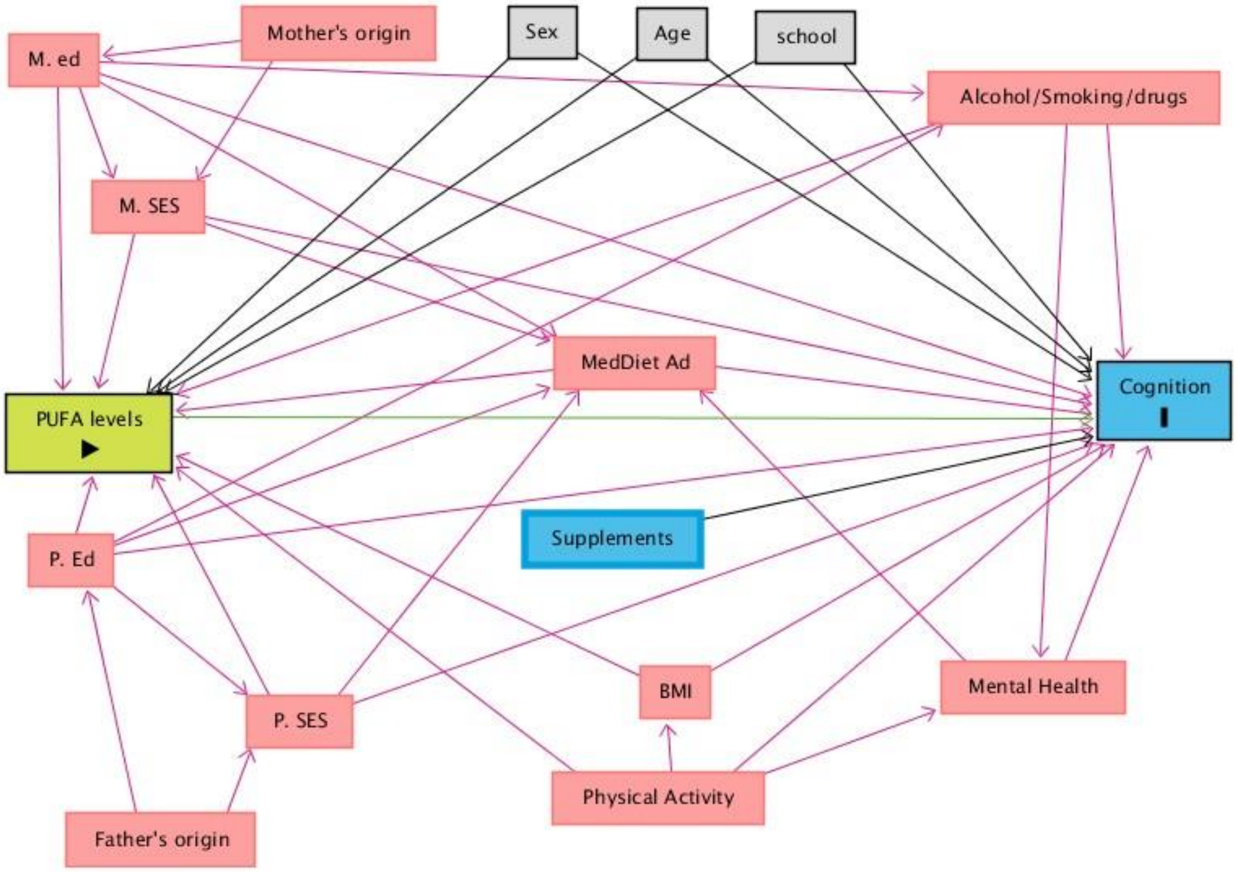

[a] Minimal sufficient adjustment sets containing Age, Sex, and center (school) for estimating the total effect of RBC PUFA levels on Cognition: Alcohol Consumption, Smoking habits, Drug consumption (Alcohol/Smoking/Drugs), Body Mass Index (BMI), Maternal and Paternal occupational social class (M. SES and P. SES), Maternal and Paternal educational level (M. ed and P. ed), Adherence to Mediterranean Diet (MedDiet Ad), Physical Activity. Drug consumption was not included in the main analyses due to low positive responses (n=15)

Supplementary Table 1. Extended descriptive characteristics of the study population

Variable	Available data (N)	Categories	N (%) / Mean ± SD
School center	332	Front Marítim	35 (10.54)
		Ernest Lluch	27 (8.13)
		Proa	35 (10.54)
		Joan Bosca	38 (11.45)
		Solc	35 (10.54)
		Sedeta	47 (14.16)
		St. Miquel	34 (10.24)
		Monserrat	35 (10.54)
		Padre Damián	25 (7.53)
		Other	21 (6.33)
Children characteristics			
Sex	332	Female	164 (49.40)
		Male	168 (50.60)
Age	327		13.84 ± 0.93
BMI	328	Kg/m^2	20.32 ± 3.23
Physical activity	310	<3 times/week	126 (40.65)
		≥3 times/week	184 (59.35)
Alcohol consumption[a]	306	No	173 (56.54)
		Yes	133 (43.46)
Smoking habits[b]	309	No	248 (80.26)
		Yes	61 (19.74)
Drug Consumption[c]	287	No	272 (94.77)
		Yes	15 (5.23)
Mental Disorder[d]	318	No	304 (95.60)
		Yes	14 (4.40)
Adherence to Mediterranean Diet[e]	305	Low	181 (59.34)
		High	124 (40.66)
Fatty Fish intake[f]	291	<4 servings/week	149 (51.20)
		≥4 servings/week	142 (48.80)
Nut intake[g]	292	<1 serving/week	153 (52.40)
		≥1 serving/week	139 (47.60)
Supplement use	305	Iron	2 (0.66)
		Calcium + VitD	2 (0.66)
		Omega 3	1 (0.33)
		Multivitamin	5 (1.64)
		Others	4 (1.31)
RBC C18:3n-3 (ALA)	332		0.11 ± 0.08
RBC C22:6n-3 (DHA)	332		3.96 ± 0.84
ANT HRT-SE[h]	321	Total Score (ms)	144.44 ± 75.40
ANT HRT[h]	322	Total Score (ms)	568.66 ± 111.28
Impulsivity Index[h]	256	Total Score (ms)	59.44 ± 155.67
Conflict response[h]	321	Total Score (ms)	67.32 ± 32.77
Orienting[h]	322	Total Score (ms)	28.79 ± 35.23
Alerting[h]	322	Total Score (ms)	30.94 ± 39.88

Mother characteristics			
Origin	316	Spain	275 (87.03)
		Other	41 (12.97)
Education	331	Lower education[i]	121 (36.56)
		University studies	210 (63.44)
Occupational social class	309	Manual	36 (11.65)
		No Manual	248 (80.26)
		Other	25 (8.09)
Father characteristics			
Origin	316	Spain	269 (85.13)
		Other	47 (14.87)
Education	329	Lower education[i]	141 (42.86)
		University studies	188 (57.14)
Occupational social class	301	Manual	49 (16.28)
		No Manual	211 (70.10)
		Other	41 (13.62)

BMI, Body Mass Index; ANT, Attention Network Test; HRT, hit reaction time; SE, standard error

[a] Defined as if they have ever consumed alcoholic beverages
[b] Defined as if they have ever smoked a cigarette, weather normal or electronic
[c] Defined as if they have ever used any drug other than alcohol and tobacco
[d] Defined as the presence of a diagnosed mental disorder
[e] A high score equals a score of 8 or more (of a total of 12)
[f] Constructed from the sum of all FFQ items related to seafood consumption (fatty fish, white fish, canned fish and fish by-products) and recategorized in two categories. One serving equals to a medium size steak, plate or portion for white and fatty fish, a small can for canned fish and two units for fish by-products (i.e. crab sticks). Categories cut-off points were selected based on statistical criteria, not based on clinical relevance
[g] Constructed from the sum of all FFQ items related to nut consumption (walnuts, almonds, hazelnuts and others) and recategorized in two categories. One serving equals to 1 handful or 6 units (just for walnuts). Categories cut-off points were selected based on statistical criteria, not based on clinical relevance
[h] Higher scores indicate worse attention performance
[i] Includes no studies, primary school and high school studies

Supplementary Table 2. Comparison of red blood cell proportions of C22:6n-3 (DHA) and C18:3n-3 (ALA) according to self-reported fish and nut consumption

Fatty acid[b]	Self-reported fish consumption[a]			Self-reported nut consumption[a]		
	<4 servings /week (n=149)	≥4 servings /week (n=142)	P value	<1 serving /week (n=153)	≥1 serving/week (n=139)	P value
C22:6n-3	3.82 (0.83)	4.22 (0.80)	<0.001	4.06 (0.81)	3.98 (0.86)	0.796
C18:3n-3	0.10 (0.01)	0.11 (0.01)	0.102	0.11 (0.01)	0.11 (0.01)	0.454

[a] Medians were used as cut-off points.
[b] Mean (SD). P value obtained by Student's t-test

Supplementary Table 3. Association between red blood cell proportions of C22:6n-3 (DHA) and C18:3n-3 (ALA) in tertiles and ANT scores on orienting network[a]

Fatty acid[b]	Orienting Network, ms							
	Minimally adjusted[c]				Fully adjusted[d]			
	N	Coef.	(95% CI)	P value	N	Coef.	(95% CI)	P value
**C22:6n-3**								
1st tertile (3.12)	106	Ref.			76	Ref.		
2nd tertile (3.89)	104	4.79	(-5.07; 14.64)	0.340	84	2.39	(-9.33; 14.10)	0.688
3rd tertile (4.75)	107	2.67	(-7.48; 12.82)	0.605	89	-1.99	(-14.12; 10.15)	0.747
Tertiles in continuous[e]	317	1.36	(-3.71; 6.43)	0.599	249	-1.07	(-7.12; 4.99)	0.729
**C18:3n-3**								
1st tertile (0.06)	104	Ref.			85	Ref.		
2nd tertile (0.09)	104	10.87	(0.45; 21.29)	0.041	90	7.04	(-5.05; 19.13)	0.252
3rd tertile (0.14)	109	5.15	(-5.73; 16.03)	0.353	74	1.80	(-10.79; 14.39)	0.778
Tertiles in continuous[e]	317	2.27	(-3.19; 7.73)	0.414	249	0.69	(-5.60; 6.99)	0.828

CI, confidence interval; Ref, reference group; Coef., β coefficients estimated by linear regression models; N, number of subjects with available data

[a] Higher scores indicate worse attention performance (orienting)
[b] Median of each fatty acid within tertile category
[c] Adjusted for: school center, age, and gender of the child
[d] Additionally adjusted for: body mass index of the child, alcohol consumption (have you ever consumed alcoholic beverages? yes/no), smoking habit (have you ever smoked a normal or electronic cigarette? yes/no), physical activity ≥3 times/week (yes/no), modified Mediterranean diet score (1-7 "low"/8-12 "high"), maternal educational level (university studies/lower education), paternal education level (university studies/lower education), maternal occupational social class (manual/non-manual) and paternal occupational social class (manual/non-manual)
[e] The p-value is P for trend

Supplementary Table 4. Association between red blood cell proportions of C22:6n-3 (DHA) and C18:3n-3 (ALA) in tertiles and ANT scores on alerting network[a]

Fatty acid[b]	Alerting Network, ms							
	Minimally adjusted[c]				Fully adjusted[d]			
	**N**	**Coef.**	**(95% CI)**	**P value**	**N**	**Coef.**	**(95% CI)**	**P value**
**C22:6n-3**								
1st tertile (3.12)	106	Ref.			76	Ref.		
2nd tertile (3.89)	104	-6.19	(-17.33; 4.94)	0.275	84	-5.20	(-18.21; 7.82)	0.432
3rd tertile (4.75)	107	-5.06	(-16.52; 6.40)	0.385	89	-7.11	(-20.59; 6.38)	0.300
Tertiles in continuous[e]	317	-2.55	(-8.28; -3.17)	0.381	249	-3.52	(-10.24; 3.21)	0.304
**C18:3n-3**								
1st tertile (0.06)	104	Ref.			85	Ref.		
2nd tertile (0.09)	104	-0.67	(-12.53; 11.19)	0.911	90	3.56	(-9.90; 17.03)	0.603
3rd tertile (0.14)	109	-1.97	(-14.36; 10.41)	0.754	74	-2.43	(-16.46; 11.59)	0.733
Tertiles in continuous[e]	317	-0.99	(-7.17; 5.17)	0.751	249	-1.38	(-8.38; 5.62)	0.698

CI, confidence interval; Ref, reference group; Coef., β coefficients estimated by linear regression models; N, number of subjects with available data

[a] Higher scores indicate worse attention performance (alerting)
[b] Median of each fatty acid within tertile category
[c] Adjusted for: school center, age, and gender of the child
[d] Additionally adjusted for: body mass index of the child, alcohol consumption (have you ever consumed alcoholic beverages? yes/no), smoking habit (have you ever smoked a normal or electronic cigarette? yes/no), physical activity ≥3 times/week (yes/no), modified Mediterranean diet score (1-7 "low"/8-12 "high"), maternal educational level (university studies/lower education), paternal education level (university studies/lower education), maternal occupational social class (manual/non-manual) and paternal occupational social class (manual/non-manual)
[e] The p-value is P for trend
